# Exploring potential predictive biomarkers through historical perspectives on the evolution of systemic therapies into the emergence of neoadjuvant therapy for the treatment of hepatocellular carcinoma

**DOI:** 10.3389/fonc.2024.1429919

**Published:** 2024-06-27

**Authors:** Chuanlei Wang, Feng Wei, Xiaodong Sun, Wei Qiu, Ying Yu, Dawei Sun, Yao Zhi, Jing Li, Zhongqi Fan, Guoyue Lv, Guangyi Wang

**Affiliations:** ^1^ Department of Hepatobiliary and Pancreatic Surgery I, General Surgery Center, The First Hospital of Jilin University, Changchun, China; ^2^ Key Laboratory of the General Surgery Health Department of Jilin Province, Changchun, China

**Keywords:** hepatocellular carcinoma, IMbrave150 trial, immunotherapy, immune-checkpoint inhibitors, neoadjuvant therapy

## Abstract

Hepatocellular carcinoma (HCC), a type of liver cancer, ranks as the sixth most prevalent cancer globally and represents the third leading cause of cancer-related deaths. Approximately half of HCC patients miss the opportunity for curative treatment and are then limited to undergoing systemic therapies. Currently, systemic therapy has entered the era of immunotherapy, particularly with the advent of immune-checkpoint inhibitors (ICIs), which have significantly enhanced outcomes for patients with advanced HCC. Neoadjuvant treatment for HCC has become a possibility—findings from the IMbrave 050 trial indicated that ICIs offer the benefit of recurrence-free survival for high-risk HCC patients post-resection or local ablation. However, only a small fraction of individuals benefit from systemic therapy. Consequently, there is an urgent need to identify predictive biomarkers for treatment response and outcome assessment. This study reviewed the historical progression of systemic therapy for HCC, highlighting notable therapeutic advancements. This study examined the development of systemic therapies involving conventional drugs and clinical trials utilized in HCC treatment, as well as potential predictive biomarkers for advanced and/or locally advanced HCC. Various studies have revealed potential biomarkers in the context of HCC treatment. These include the association of dendritic cells (DCs) with a favorable response to neoadjuvant therapy, the presence of enriched T effector cells and tertiary lymphoid structures, the identification of CD138+ plasma cells, and distinct spatial arrangements of B cells in close proximity to T cells among responders with locally advanced HCC receiving neoadjuvant cabozantinib and nivolumab treatment. Furthermore, pathological response has been associated with intratumoral cellular triads consisting of progenitor CD8+ T cells and CXCL13+ CD4+ T helper cells surrounding mature DCs in patients receiving neoadjuvant cemiplimab for resectable HCC. Despite no widely recognized predictive biomarkers for HCC individualized treatment, we believe neoadjuvant trials hold the most promise in identifying and validating them. This is because they can collect multiple samples from resectable HCC patients across stages, especially with multi-omics, bridging preclinical and clinical gaps.

## Introduction

Hepatocellular carcinoma (HCC), which comprises the vast majority of liver cancer cases and fatalities, ranked as the sixth most frequently diagnosed cancer and third leading cause of cancer-related mortalities, according to 2020 estimates (1). Currently, the hepatitis B virus (HBV) and hepatitis C virus (HCV) are the primary global risk factors for HCC (2). However, among all HCC cases, the proportion of HBV- and HCV-related cases is declining, while there is an increasing trend in HCC cases caused by metabolic dysfunction-associated fatty liver disease (MAFLD). Metabolic syndrome, obesity, type II diabetes, and non-alcoholic fatty liver disease are significant risk factors contributing to the emergence of MAFLD-related HCC (3).

Due to a lack of symptoms in early-stage patients, HCC is often diagnosed at an advanced stage, which diminishes the possibility of curative treatment options (4). Furthermore, there is a significant risk of tumor recurrence after curative treatment, as evidenced by reported 5-year recurrence rates ranging from 40% to 70% (5). Systemic therapy plays a pivotal role in treating advanced HCC. In this context, the historical progression of systemic therapy for HCC, spanning from cytotoxic chemotherapy to immunotherapy, was reviewed, highlighting the notable therapeutic advancements that were achieved. However, a significant proportion of patients with advanced HCC experience therapeutic resistance and disease progression after receiving treatment. Consequently, there is an urgent need to identify predictive biomarkers for treatment response and outcome assessment. This will facilitate the selection of patients who are most likely to derive benefits from specific therapeutic approaches, thereby optimizing treatment outcomes, minimizing avoidable toxicities, and conserving healthcare resources.

There are two approaches to obtaining predictive biomarkers. The first relies on inferring predictive biomarkers based on the expression levels of therapeutic targets, which are subsequently validated. However, due to a substantial gap between forward translation results and clinical application over the past few decades, this approach has encountered significant challenges and can be considered relatively unsuccessful. The second involves obtaining samples, such as blood, urine, or tissue specimens, from patients who have demonstrated responsiveness to a treatment. By comparing these samples with those from non-responsive patients, predictive biomarkers can be identified.

Nevertheless, the exploration of predictive biomarkers is greatly hindered by several factors, including the fact that HCC diagnosis does not necessarily rely on histopathology; systemic therapy is primarily administered to late-stage patients; tumor biopsy collection during trials is lacking; and only a limited number of patients undergo such regimens at individual clinical sites. As a result, there is currently no universally recognized set of predictive biomarkers for HCC. However, the advent of the immunotherapy era, particularly the upcoming preoperative neoadjuvant period, enables the obtainment of on-treatment longitudinal biopsy samples. This, coupled with well-designed biomarker-driven preoperative neoadjuvant trials and the rapid development of technologies such as single-cell genomics, epigenomics, transcriptomics, spatial transcriptomics, and multi-omics sequencing, holds great potential for advancing the development of novel treatment strategies and achieving better identification of patients who are likely to benefit from immunotherapy.

## Era of cytotoxic chemotherapy

In situations where no other treatment options are available, cytotoxic chemotherapy is considered to be a viable approach, despite the lack of substantial evidence supporting its purported survival benefits (6). Notably, doxorubicin (DOX), one of the earliest chemotherapeutic drugs utilized for HCC treatment, belongs to this category (7). Whether used as monotherapy or combination therapy, the objective response rate (ORR) of HCC to DOX treatment does not surpass 20%. Nevertheless, this did not hinder its status as a first-line chemotherapeutic agent for several years until the advent of sorafenib.

Subsequent advancements in cytotoxic chemotherapy have predominately focused on comparing the treatment efficacy of alternative therapies with DOX. However, the emergence of several DOX derivatives (8–10) designed to reduce cardiotoxicity did not significantly improve therapeutic outcomes. Similar situations were observed with 5-fluorouracil (11) and its derivatives (12, 13), as well as typical platinum-based chemotherapeutic agents (14, 15). However, the emergence of 5-fluorouracil, leucovorin, and oxaliplatin (FOLFOX4) (16) led to notable results in terms of overall survival (OS) among the Chinese population, despite the absence of a significant survival benefit. Consequently, the National Comprehensive Cancer Network (NCCN) 2021 guidelines recommend the FOLFOX4 regimen for advanced or metastatic HCC patients with elevated bilirubin levels. Although the latest versions of the NCCN guidelines in 2023 (17) and European Society for Medical Oncology guidelines in 2021 (18) no longer include cytotoxic chemotherapy, FOLFOX4 was administered to patients with locally advanced or metastatic HCC before sorafenib became available.

Palliative care may still be recommended in guidelines for economically underdeveloped regions worldwide, as exemplified by the Chinese Health Commission’s Guidelines for the Diagnosis and Treatment of Primary Liver Cancer (2022 Edition) (19). Cytotoxic chemotherapy has limited efficacy for locally advanced or metastatic HCC, as only a small fraction of patients experience significant palliative effects, and the toxicity associated with chemotherapy often outweighs its potential benefits. Moreover, patients with advanced HCC frequently present with severe cirrhosis, poor performance status, and various complicating factors, such as portal vein tumor thrombosis, which contribute to an exceedingly poor prognosis when subjected to cytotoxic chemotherapy (20).

During the era of cytotoxic chemotherapy, the investigation of predictive biomarkers for HCC prognosis was constrained. In a study conducted by Patt et al. (21), 31% (five out of 16) of HCC patients with low serum alpha-fetoprotein (AFP) levels (≤50 ng/mL) and those with tumors involving 50% or less of the liver demonstrated a sustained partial response to the regimen of intravenous 5-FU and subcutaneous rIFN-alpha-2b. Conversely, Choi et al. (22) reported that serum AFP levels did not demonstrate a correlation with response to doxorubicin cytotoxic chemotherapy. Instead, HbsAg-positive patients showed a significantly higher likelihood of response, highlighting the absence of a universally recognized predictive biomarker.

## Era of multitarget kinase inhibitors

**Figure 1:**
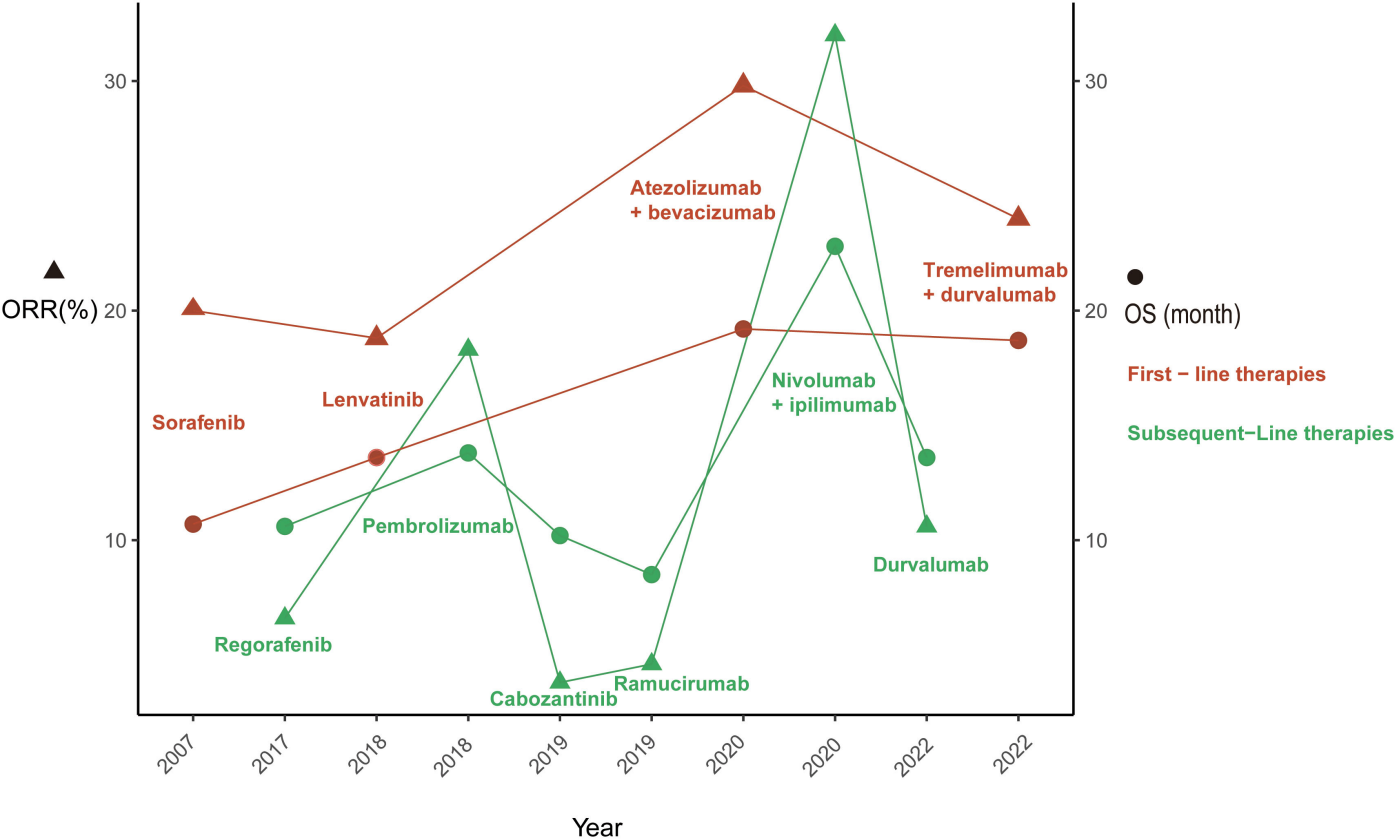
This figure illustrates the evolution U.S. Food and Drug Administration approved therapies for advanced hepatocellular carcinoma. ORR, objective response rate; OS, overall survival.

Sorafenib, an oral multitarget tyrosine kinase inhibitor (TKI) with anticancer activity and safety benefits that have been corroborated by real-world evidence, emerged as a groundbreaking treatment in 2007. Its remarkable performance in the SHARP (23) and ORIENTAL (24) trials led to U.S. Food and Drug Administration (FDA) approval of its use as the first-line treatment for unresectable HCC in patients without prior systemic therapy. Subsequently, a significant focus was placed on sorafenib, with numerous trials comparing and evaluating novel first-line agents against it to enhance the prognosis of HCC patients. Following the FDA’s approval of lenvatinib (25), regorafenib (26), cabozantinib (27), and ramucirumab (28) for first- or second-line HCC treatment due to their clinical and survival benefits (**Figure 1**), several trials were conducted comparing these novel agents, including sunitinib (29), brivanib (30), cediranib (31), linifanib (32), and dovitinib (33), with sorafenib. However, none of these trials achieved the predefined primary endpoints. Nonetheless, sorafenib used as the first-line monotherapy for advanced HCC increased the median OS from 10.7 months (SHARP trial) to 14.7 months (CheckMate-459), further consolidating its position as a first-line treatment.

Targeted therapy with sorafenib has demonstrated significant potential for enhancing the prognosis and OS of patients with advanced HCC. Consequently, since its inception, there has been substantial interest in identifying predictive biomarkers for HCC patients undergoing sorafenib treatment, although no universally recognized predictive biomarkers have been identified thus far.

Sorafenib exerts its therapeutic effects by inhibiting the extracellular-signal-regulated kinase (ERK)-independent activities of Raf1, thereby causing tumor cell apoptosis, and suppressing the activity of receptor tyrosine kinases (RTKs) such as vascular endothelial growth factor receptors (VEGFR) 1, 2, and 3, as well as platelet-derived growth factor receptor beta (PDGFR-β), thereby inhibiting tumor angiogenesis and promoting apoptosis (23, 34, 35). Moreover, sorafenib hampers the tumor cell proliferation involved in tumorigenesis *in vitro* by targeting other RTKs, including c-Kit, Flt-3, and RET. Consequently, the first predictive biomarker that comes to mind is the therapeutic target of sorafenib. However, since sorafenib is indicated for patients with advanced HCC, obtaining HCC tissue samples for exploring predictive biomarkers is challenging, which has resulted in limited studies, most of which relied upon blood rather than tissue samples. Results from *in vitro* experiments on HCC cell lines are limited, as the inhibitory effect of sorafenib on tumor growth involves inhibiting tumor angiogenesis.

Kim et al. conducted a study involving 220 HCC patients treated with sorafenib, in which they examined the expression of seven actionable genes (VEGFR2, PDGFR-β, c-Kit, Raf1, EGFR, mTOR, and FGFR1) in tumor tissues compared to those in adjacent normal tissues. They found that the combination of mTOR, VEGFR2, c-Kit and c-RAF was the most significant predictor of response to sorafenib, resulting in a tumor response rate of 15.6% (36). Chu et al. performed immunohistochemical analyses of biopsy tissues from 93 HCC patients treated with sorafenib, focusing on the expressions of VEGFR-2, PDGFR-β, and c-Met. Their findings indicated that high expression of PDGFR-β was associated with poor prognosis, while elevated expression of c-Met may predict the therapeutic efficacy of sorafenib in HCC patients (37). Abou-Alfa et al. analyzed immunochemically stained phosphorylated ERK (pERK) levels in pretreatment biopsies and blood-cell RNA expression in 137 HCC patients treated with sorafenib, discovering that patients with higher baseline pERK expression had a longer median time to progression (TTP) (38). However, Personeni et al. found that pERK expression levels did not influence TTP. Their study involving 44 patients with advanced HCC who received sorafenib showed that the expression levels of myeloid cell leukemia-1 (Mcl-1) and pERK were associated with reduced OS through the expression of Mcl-1, activated/phosphorylated extracellular signal-regulated kinase (pERK) 1/2, and activated/phosphorylated AKT (pAKT) in pretreatment tumor specimens (39). El Shorbagy et al. conducted a prospective, randomized study involving 80 advanced measurable HCC patients who received either sorafenib plus metformin (arm A) or sorafenib alone (arm B). They evaluated the plasma and tissue levels of VEGF and HIF-1α and found that low VEGF and HIF-1α plasma levels were significantly associated with better treatment response and longer OS, whereas high expressions of VEGF and HIF in HCC tissue were linked to poor treatment outcomes and shorter OS (40).

Single nucleotide polymorphisms (SNPs) in VEGF and VEGFR genes have shown correlations with progression-free survival (PFS) and OS. For instance, the study conducted by Scartozzi et al. demonstrated that VEGF-A rs2010963 and VEGF-C rs4604006 were independent factors affecting PFS and OS when analyzing 148 samples (tumor or blood samples) from HCC patients treated with sorafenib and tested for VEGF-A, VEGF-C, and VEGFR-1, -2, and -3 SNPs (41). MiRNA-related research has also yielded significant findings. Vaira et al. observed that high levels of miR-425–3p were associated with TTP and PFS in their investigation of miRNA expression in tumor and cirrhotic liver biopsies from 84 HCC patients treated with sorafenib (42). Similarly, Gyöngyösi et al. discovered that elevated miR-224 expression was correlated with increased PFS and OS, based on their analysis of total RNA extracted from diagnostic fine-needle aspiration biopsy cytological smears of 20 advanced-stage HCC patients prior to sorafenib treatment (43).

Although plasma biomarkers are considered the most suitable candidates for evaluating sorafenib efficacy, only a limited number of trials, such as the SHARP trial, have produced results with borderline significance. One notable example is AFP, which is secreted by approximately 50% of all HCCs. The SHARP trial revealed that high baseline AFP plasma levels (>200 ng/mL) had a negative impact on OS (23). This finding was recently validated by a pooled analysis combining data from the SHARP trial and the Asia–Pacific trial, where an AFP level >400 ng/mL was identified as a prognostic factor for poorer OS by Bruix et al. (44). Moreover, the SHARP trial identified angiopoietin-2 (Ang-2) as the only biomarker prognostic for both OS and TTP. Low baseline plasma concentrations of Ang-2 and VEGF were associated with improved median survival. However, none of these biomarkers significantly predicted response to sorafenib (23). Similar results were reported by Miyahara et al., who found that high baseline levels of serum cytokines, including Ang-2, granulocyte colony-stimulating factor (G-CSF), hepatocyte growth factor (HGF), and leptin were correlated with poor treatment effects of sorafenib in HCC patients (45). Tsuchiya et al. demonstrated that a decrease in plasma VEGF concentration at 8 weeks after initiating sorafenib treatment may predict favorable OS based on serial measurements in 63 patients with advanced HCC before and during sorafenib treatment (46).

Following sorafenib, lenvatinib is the only multitarget tyrosine kinase inhibitor (TKI) currently approved by the U.S. FDA for the first-line treatment of unresectable advanced HCC. Predictive biomarkers for lenvatinib have primarily focused on its main targets, including VEGFR 1–3, FGFR 1–4, PDGFRα, as well as proto-oncogenes RET and KIT. Lenvatinib exhibits a distinct kinase inhibitory profile and binding mode compared to sorafenib (47–51). Furthermore, recent research indicates that lenvatinib activates innate and acquired anti-tumor immunity (52). In an analysis involving 407 patients conducted by Finn et al., higher baseline serum levels of VEGF, FGF21, and Ang-2 were found to be potentially prognostic for shorter OS. The study also observed a decrease in Ang-2 levels from baseline specifically in the lenvatinib group, suggesting that TIE-2 signaling was influenced by lenvatinib but not sorafenib. Additionally, gene expression analysis of tumor tissue from 58 patients revealed that the enrichment of VEGF and FGF pathways was associated with improved OS in the lenvatinib arm compared to the intermediate group. Lenvatinib-treated patients in the Wnt subgroup, utilizing 13 canonical cancer pathways including β-catenin and DNA-repair pathways, exhibited different OS outcomes (53). Similar findings were reported by Chuma et al., who conducted a prospective cohort study involving 101 patients and found that increases in serum FGF19 levels and decreases in serum Ang-2 levels early in treatment were associated with a response in patients receiving lenvatinib and were correlated with longer PFS (54). In a study by Liu et al., which included 46 unresectable advanced HCC patients with AFP levels ≥20 ng/mL, it was observed that early responders to lenvatinib based on AFP levels achieved significantly higher ORR and disease control rates compared to non-responders (55).

The results of other second-line treatments, such as the TKIs regorafenib, cabozantinib, and ramucirumab, have not yielded eye-catching predictive biomarker outcomes. Please refer to **Table 1** for further details.

**Table 1:** Targets, receptors, and potential predictive biomarkers for the response of HCC patients to targeted therapy (excluding results from preclinical animal models and HCC cell lines).

Multikinase inhibitor	Target receptors	Potential predictive biomarkers	References
Sorafenib	VEGFR1–3, PDGFR-β, c-Kit, Flt-3, RET	AFP, mTOR, VEGFR2, c-Kit, c-RAF, PDGFR-β, Mcl-1, pERK, VEGF-A, VEGF-C, miR-425–3p, miR-224, Ang-2, HGF/c-Met, G-CSF, leptin	(23, 36–38) (39–44) (45, 46)
Lenvatinib	VEGFR1–3, FGFR1–4, PDGFRα, RET, c-Kit	AFP, HIF-1 α, VEGF, FGF21, Ang-2, FGF19, NLR	(53–56)
Regorafenib	VEGFR1–3, FGFR1, PDGFR-β, KIT, RET, B-RAF	AFP, LAP TGF-β1, Ang1, cystatin B, LOX-1, miP-1α, miR30A, miR122, miR125B, miR200A, miR374B, miR15B, miR107, miR320B, miR645, SII	(57–59)
Cabozantinib	VEGFR2, MET, AXL, RET, FLT3, KIT	AFP	(60, 61)
Ramucirumab	VEGFR2	AFP	(62)

AFP, alpha-fetoprotein; NLR, neutrophil-to-lymphocyte ratio; SII, systemic immune-inflammation index.

## Era of immunotherapy

Subsequent to multitarget kinase inhibitor (MKI) treatment, immunotherapy has shown clinical efficacy and a more favorable toxicity profile in the management of advanced HCC, with a particular emphasis on ICIs. The primary targets for ICIs encompass cytotoxic T lymphocyte-associated molecule-4 (CTLA-4), programmed cell death receptor-1 (PD-1), and programmed cell death ligand-1 (PD-L1). In May 2020, the FDA approved atezolizumab, an anti-PD-L1 antibody, and the VEGF-neutralizing antibody bevacizumab for the first-line treatment of patients with unresectable or metastatic HCC (**Figure 1**), marking the beginning of the era of immunotherapy for advanced HCC. The renowned IMbrave150 trial demonstrated a median OS of 19.2 months in the atezolizumab plus bevacizumab arm compared to 13.4 months in the sorafenib arm (63). In terms of ORR, atezolizumab plus bevacizumab achieved 30% compared to 11% with sorafenib (64). Furthermore, pembrolizumab, an anti-PD-L1 antibody, and nivolumab plus ipilimumab, a combination of anti-PD-L1 and anti-CTLA-4 antibodies, have also been approved for the first-line treatment of patients with locally unresectable or metastatic HCC (65, 66).

The theoretical foundation for utilizing ICIs and anti-VEGF antibodies in HCC treatment rests upon three key concepts: the “cancer-immunity cycle” theory (67, 68), “normalization cancer immunotherapy” (69), and “normalizing tumor vasculature” (70), which is alternatively referred to as “normalization of the tumor microenvironment (TME)” (71). When exploring predictive biomarkers for HCC, two prominent candidates come to mind: PD-L1 and tumor-infiltrating lymphocytes (TILs). PD-L1 is expressed by various cell types, including cancer cells, macrophages, B cells, DCs, and tumor stroma. In some cancer types, such as non-small-cell lung cancer, intratumoral PD-L1 staining appears to be somewhat correlated with outcomes. However, this correlation is not true for HCC. In the CheckMate 040 trial, tumor cell PD-L1 expression at 1% or higher was associated with improved OS (*p*=0.032) for nivolumab in the overall patient population, including both sorafenib-treated and sorafenib-naive individuals (65). However, the same conclusion was not reached in the CheckMate 459 trial (72). This discrepancy can be attributed to intratumoral heterogeneity in PD-L1 expression, which is often missed in small-specimen biopsies. Moreover, different antibodies used for PD-L1 detection exhibit varying affinities and specificities, whereas the use of different detection assays, inter-assay and/or inter-observer heterogeneity, and non-standardized criteria and cut-offs for positivity may also contribute to diverse findings (73–75).

As previously mentioned, obtaining tumor tissues from patients with advanced or metastatic HCC undergoing ICI treatment is highly challenging. However, acquiring peripheral blood samples is comparatively easier and less invasive, making them more feasible for exploring biomarkers. Serum levels of soluble PD-L1 (sPD-L1) appear to be correlated with outcomes in patients with HCC, in which elevated levels of sPD-L1 are indicative of poorer prognosis; however, the results have been inconsistent (76–81). Intratumoral TILs are considered key players in immunotherapy. In the CheckMate 040 trial, Sangro et al. conducted an analysis using immunohistochemistry and RNA sequencing, which revealed increased levels of CD3 and CD8 TILs, while macrophages exhibited a non-significant trend towards improved OS (82). Another study by Ng et al., which analyzed 49 patients with advanced HCC treated with an immune checkpoint blockade, assessed CD38 expression through immunohistochemistry (IHC), multiplex immunohistochemistry/immunofluorescence (mIHC/IF), and multiplex cytokine assays. This study demonstrated that a high proportion of CD38+ cells responded to the immune checkpoint blockade and was associated with superior median PFS and OS (83). Another commonly studied predictive biomarker is the level of AFP in peripheral blood. Zhu et al. conducted analyses using data from the GO30140 study and the IMbrave150 trial, identifying that changes in AFP cutoffs of ≥75% and ≤10% from baseline at 6 weeks could be utilized to distinguish between responders and disease control. Moreover, both AFP cutoffs were associated with extended OS and PFS, especially in patients with hepatitis B virus etiology (84).

Several other peripheral blood-derived predictive biomarkers exhibited similar positive effects. For instance, the neutrophil-to-lymphocyte ratio and platelet-to-lymphocyte ratio were correlated with decreased OS and PFS, while not affecting ORR or disease control rates (85–92). Long-term responders who received regorafenib plus nivolumab immunotherapy showed enrichment in MKI67+ proliferating CD8+ T cells and a higher probability of M1-directed monocyte polarization in peripheral blood mononuclear cells (93). Additionally, in the setting of atezolizumab/bevacizumab (Atezo/Bev) combination immunotherapy for unresectable HCC, patients with elevated cell-free tumor DNA (cfDNA) levels displayed notably diminished overall response rates and shorter progression-free survival as well as overall survival compared to those with low cfDNA levels. While the presence or absence of circulating tumor DNA (ctDNA) did not forecast therapy efficacy, the identification of a TERT mutation in ctDNA and AFP levels ≥400 ng/mL independently served as indicators of unfavorable overall survival outcomes associated with the therapy (94). Circulating cfDNA with the initial CXCL9 (<333 pg/mL) accurately forecasted early progression in patients undergoing immunotherapy with the atezolizumab and bevacizumab combination (95). PD-L1+ circulating tumor cells (CTCs) were accurate differential cancer staging biomarkers between early- and advanced-stage HCC and were associated with immunotherapy response (96). Furthermore, in a small subset of 10 patients with HCC who were receiving anti-PD-1 antibodies, the presence of PD-L1+ CTCs was predominantly observed in patients with locally advanced or metastatic disease and independently prognosticated OS (97).

Intra-tumor predictive biomarkers provide a better reflection of the actual conditions within the tumor when compared to peripheral blood-derived predictive biomarkers. The immune environment and immune processes in the tumor are highly complex and dynamically change during different stages of disease progression. Therefore, it is imperative to explore predictive biomarkers for HCC within the TME in order to gradually understand the intricacies of HCC and its immune response. The emergence of rapidly evolving single-cell sequencing technologies, encompassing methods such as single-cell genome, epigenome, transcriptome, and multi-omics sequencing, has facilitated this exploration. Zheng et al. conducted single-cell RNA sequencing on 5,063 T cells isolated from the peripheral blood, tumor, and adjacent normal tissues of six HCC patients. They discovered the presence of CD4+ T cells within the tumor and the expansion of regulatory T cells (Tregs) from adjacent normal tissue into the TME. Furthermore, they observed the transformation of effector CD8+ T cells into exhausted CD8+ T cells, with CD8+FOXP3+ regulatory T cells specifically existing in the HCC microenvironment and promoting this transition. These cells demonstrated a transitional state known as pre-exhaustion CD8+ T cells, which are characterized by the expression of GZMB, GNLY, and KLRG1 markers. It is speculated that the antibody blockade of the PD-1 pathway primarily acts on these pre-exhaustion CD8+ T cells. Additionally, they identified cytolytic CD4+ T cells (CXCR6, TBX21, and CXCR3) and mucosal-associated invariant T cells (MAIT) as potential therapeutic targets. Moreover, they found a higher proportion of exhausted CD8+ T cells in late-stage compared to early-stage HCC, suggesting a correlation between disease progression and further deterioration of systemic immune status (98). Zhang et al. discovered that LAMP3+ DCs expressed maturation markers (LAMP3, CD80, and CD83) and the migration marker CCR7. These LAMP3+ DCs originated from cDC2 and cDC1 through maturation processes. They exhibited a unique capacity to regulate lymphocytes in the TME through cross-talk and migrate to lymph nodes (LNs). Importantly, LAMP3+ DCs were correlated with dysfunctional T cells. The researchers also identified higher levels of the lymphocyte recirculation chemokines CCL19 and CCL21 in HCC tumors compared to adjacent liver tissue, which promoted the migration of LAMP3+ DCs from the tumor to LNs (99).

**Figure 2:**
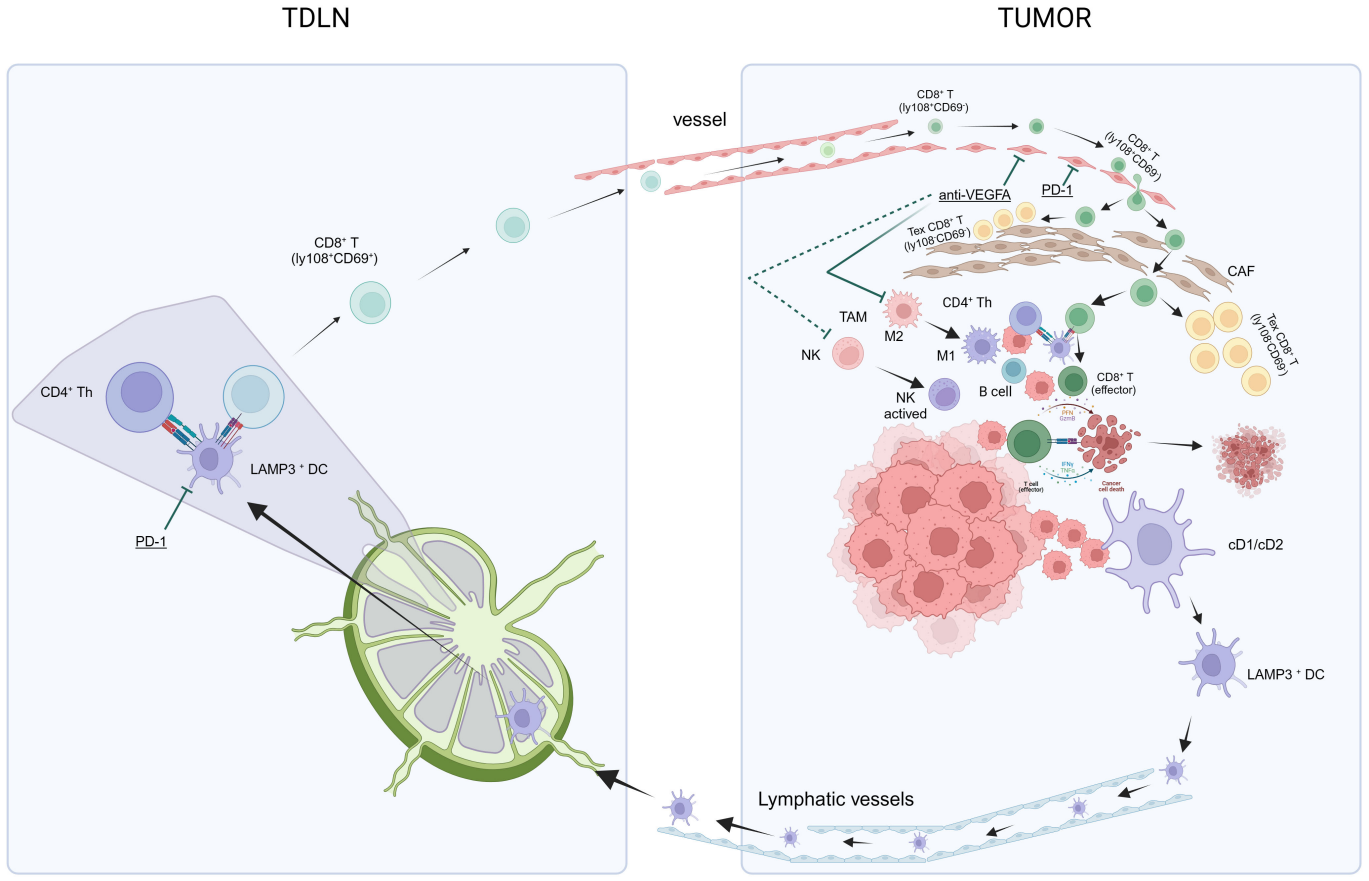
In summary, the theory of anti-PD-L1 and anti-VEGF antibody treatments in HCC aims to achieve “normalization of the TME” through a method of “normalizing the tumor vasculature.” Manipulating the anti-VEGF antibody restores vascular permeability, enhances perfusion, improves oxygenation, and reduces the acidic milieu of the TME. This mitigates immune suppression and initiates a transition: The conversion of TAMs from an M2-type immune suppression state to an M1-type anti-tumor state, and the activation of NK cells into an anti-tumor state. Concurrently, the anti-PD-L1 antibody facilitates normalized T-cell adhesion within the vascular endothelium, thereby enabling T-cell infiltration into the TME. Alongside this, other immune and non-immune cells that have regained functionality via the anti-PD-L1 antibody contribute to “normalization of the tumor microenvironment,” which intensifies the “cancer-immunity cycle,” culminating in “normalized cancer immunotherapy.” CAF, cancer-associated fibroblasts; HCC, hepatocellular carcinoma; NK, natural killer; PD-l1, programmed cell death ligand-1; TDLN, tumor-draining lymph nodes; TAMs, tumor-associated macrophages; TME, tumor microenvironment; VEGF, vascular endothelial growth factors.

Dammeijer et al. elucidated that PD-L1+ conventional DCs (cDCs), rather than macrophages within tumor-draining lymph nodes (TDLNs) or the tumor itself, could activate early-effector T cells. As such, TDLNs have emerged as potential primary targets for PD-1/PD-L1 checkpoint blockade, ultimately amplifying the induction of anti-tumor T cells (100). Subsequently, utilizing murine tumor models, Prokhnevska et al. demonstrated that tumor-specific CD8+ T cells (PD1+ CD45RA− TCF1+) were activated in TDLNs but did not exhibit an effector phenotype until migration into the tumor occurred. Furthermore, additional co-stimulation from antigen-presenting cells facilitated effector differentiation (101). Beltra et al. established a four-cell-stage developmental framework for exhausted CD8+ T (Tex) cells. They identified two TCF1+ progenitor subsets, one characterized as tissue-restricted and quiescent, while the other was more accessible through the bloodstream. These subsets gradually lost TCF1 expression as they underwent division and transitioned into a third intermediate Tex subset. The intermediate subset exhibited partial re-engagement of effector functions and demonstrated an increased response to the PD-L1 blockade; however, it eventually transformed into a fourth subset characterized as terminally exhausted (102). Chu et al. further detailed the roadmap of T cell exhaustion, which elucidated changes in key transcription factors (PD1, Cxcr5, Tcf7, T-bet, Tox, Ki67) throughout this process (103). The aforementioned research results contributed to filling in the gaps in the previously proposed “cancer-immunity cycle” theory (67, 68), and enhanced our comprehension of the activation, development, and exhaustion processes of CD8+ T cells (**Figure 2**), thus potentially leading to the discovery of target genes that are amenable to modulation.

The initial activated tumor-specific CD8+ T cells in TDLNs enter the HCC TME via the bloodstream and subsequently undergo induction into non-functional exhausted CD8+ T cells. Questions surrounding this are whether this process can be prevented, and if it is possible to achieve “normalization of the TME” (71). There is a crucial need to normalize the structure of the TME, which would require the utilization of anti-VEGFA agents to rectify abnormal changes in blood vessels within HCC. This approach aims to achieve “normalization of the TME” (71) by normalizing the tumor vasculature. The administration of low doses of anti-VEGF monoclonal antibodies can normalize blood vessels, reprogram the TME from immunosuppression to potentiation, specifically polarize macrophages towards an M1-like (immunostimulatory) phenotype, and facilitate the trafficking and activation of cytotoxic T lymphocytes (CTLs) in experimental tumors, thereby enhancing the efficacy of ICIs (104, 105). Unfortunately, effective normalization, referred to as the “normalization window,” is time-limited. The duration of this window period for HCC remains unknown and varies among individuals, posing a challenge to its clinical application (71). Additionally, various other approaches are actively under investigation to achieve vascular normalization, including targeting Ang-Tie signaling, oncogenic signaling in cancer cells, and even CD4+ T-cells (106).

In addition, a tumor barrier within the TME consists of numerous stromal cells that may contribute to the failure of tumor rejection. One such factor is the tumor-necrosis factor superfamily member known as LIGHT (TNFSF-14), which serves as a ligand for the lymphotoxin-β receptor expressed on stromal cells and the herpes viral entry mediator expressed on T cells. Targeting LIGHT in treatment not only recruits naive T cells but also selectively expands tumor antigen-specific T cells within tumor tissues, thereby fostering a T cell-inflamed microenvironment and overcoming tumor resistance to checkpoint blockade therapy (107–109).

Therefore, the exploration of predictive biomarkers for HCC and immunotherapy stems from the aforementioned investigation into the HCC TME. Delving into further details is beyond the scope of this discussion. Please refer to **Table 2** for additional information on the exploration of predictive biomarkers in the HCC TME through immunotherapy.

**Table 2:** Potential targets and predictive biomarkers within the HCC TME for US FDA-Approved First-Line and Subsequent-Line immunotherapy treatments.

Biomarkers	Agent	Benefits	Source	Reference
Pre-existing immunity (CD8 and CD4 T cells, Tregs, B cells and dendritic cells)	Atezolizumab, bevacizumab	Associated with better response and longer PFS	Tissue/bulkRNA-seq	(110)
TIB, consisting of SPP1+ macrophages and CAFs positioned in proximity to the tumor boundary	Anti-PD-1	Significantly higher in non-responders than in responders	Tissue/scRNA-seq and ST	(111)
TIH	Durvalumab, tremelimumab, pembrolizumab	Higher cluster number associated with substantially shorter survival	Tissue/scRNA-seq	(112)

CAFs, cancer-associated fibroblasts; PFS, progression-free survival; scRNA-seq, single-cell RNA-sequencing; TIB, tumor immune barrier; TIH, intratumor heterogeneity.

## Era of preoperative neoadjuvant therapy

The preoperative neoadjuvant period, which began with the exploration of MKIs (113), is not solely a progression of the immunotherapy era. However, the advent of immunotherapy has greatly benefited an increasing percentage of HCC patients. As the paradigm shifts towards reverse and forward translation research, neoadjuvant therapy for HCC has become a possibility. While organoids or organotypic explant cultures can partially capture the dynamics of tumor-associated T cells or the “cytotoxic revival” of T cells after immunotherapy (114), they cannot fully replicate the impact of the extratumoral environment on tumor immunity. Animal models remain crucial for dynamically analyzing the multidimensional processes involving multiple organs and diseased tissues. Disregarding the underwhelming success rate of current translational medicine based on animal models would be unwise. Therefore, a combination of reverse and forward translation in fundamental and translational immunology research holds great potential for improving the clinical success rates of immunotherapy (115–118).

Immunotherapy of localized tumors necessitates systemic involvement. This requirement is evident in the “cancer-immunity cycle” (67, 68), which involves the participation of tumor-draining lymph nodes (TDLNs) and peripheral blood circulation (**Figure 2**). Tumor eradication requires immune activation in the periphery. For instance, Spitzer et al. discovered that early post-therapy upregulation of PD-L1 protects distal tumors from systemic immunity (119). Additionally, Reticker-Flynn et al. found that LN colonization induces extensive alterations in the local immune repertoire in a syngeneic melanoma mouse model of LN metastasis, where LN metastases induce antigen-specific Tregs that promote distant metastasis (120). In the global, open-label, phase 3 IMbrave050 trial investigating adjuvant atezolizumab plus bevacizumab versus active surveillance in high-risk HCC patients, Qin et al. observed significantly improved recurrence-free survival with adjuvant atezolizumab plus bevacizumab (121). Thus, the premise of immunotherapy for HCC involves extending immunotherapies to early HCC cases with a risk of early recurrence, resectable locally advanced HCC, or the adjuvant setting, where immunotypes may be less restrictive and potentially more adaptable. Adopting a “reverse translational approach” to obtain longitudinal samples from patients can provide insights into evolving immune responses in the TME. Immune profiling of pretreatment and on-treatment longitudinal biopsy samples can offer crucial information regarding changes in relevant targets within specific patient cohorts. Correlative data linking clinical outcomes with target expressions can elucidate the functional impact of these targets. Subsequently, these targets can be evaluated in immunocompetent preclinical models to guide rational combination therapy strategies in future clinical trials (122).

Neoadjuvant immunotherapy offers numerous advantages. It can induce T cell expansion and is particularly beneficial at earlier stages of cancer, when T cell function is less compromised. Moreover, the routine biopsy of surgical specimens allows for convenient assessment of treatment effects. Additionally, immunotherapy has the potential to reduce tumor size before surgery, potentially enhancing surgical outcomes (123). Neoadjuvant checkpoint inhibitors can effectively promote the *de novo* induction of T cell-mediated immunity by recognizing primary tumor antigens, expanding pre-existing antitumor T cells, and fostering the development of a diverse repertoire of tumor-specific T cells more efficiently than in the adjuvant setting, after tumor removal. These tumor-specific memory T cells exhibit superior capability in recognizing micrometastases that are not visible to the naked eye, thereby reducing the risk of recurrence following radical surgery (124, 125).

For the implementation of preoperative ICI neoadjuvant treatment in HCC, reliable predictive biomarkers are essential for identifying suitable patient groups. However, as research on the TME in HCC deepens, it becomes evident that relying on a single biomarker is insufficient for predicting treatment response. The updated “cancer-immunity cycle” in 2023 introduced the concept of local small cycles to complement multi-cell cooperation within the TME. Specifically, T cells encounter antigen-presenting cells, particularly DCs, dispersed within the tumor parenchyma, tumor-associated lymphoid aggregates, or morphologically identifiable tertiary lymphoid structures (TLSs), leading to their expansion, differentiation, and subsequent direct killing of tumor cells (68).

Therefore, by comparing the “cancer-immunity cycle” (67, 68), “normalization cancer immunotherapy” (69), and “normalizing tumor vasculature” (70)—also referred to as “normalization of the TME” (71)—we aim to explore predictive biomarkers for HCC. Neoadjuvant research in HCC has emerged as the most favorable approach for investigating potential biomarkers. Preoperative neoadjuvant clinical trials for HCC have been approved and are currently underway. Please refer to **Table 3** for further details. From these clinical trials, it is evident that a series of key factors must be considered to ensure the scientific validity and clinical relevance of research outcomes. Firstly, these clinical trials must ensure an adequate sample size, as studies with small sample sizes may lack representativeness, thus limiting the generalizability of results. Secondly, patient selection and exclusion criteria affect the generalizability of research results. It is essential to consider the impact of different etiologies on the effectiveness of neoadjuvant therapy in HCC patients. Long-term follow-up and survival data are crucial for a comprehensive assessment of treatment efficacy. Furthermore, stringent randomization, blinding, and control group setup form the basis for assessing bias risks and ensuring the quality of the study design. Transparency regarding funding sources and researchers’ declarations is essential for identifying potential conflicts of interest and reporting biases. Considering these factors collectively can provide a robust evidence base for clinical practice and guide future research directions. Although there are limited published results from preoperative neoadjuvant clinical trials in HCC, the credibility of these results is substantial. We will provide a summary of the findings that have been published thus far.

**Table 3:** Neoadjuvant and/or adjuvant treatment clinical trials for hepatocellular carcinoma.

NCT Number	Agent	Perioperation	Study Status	Conditions	Phases	Study Type	Start Date
NCT03510871	Nivolumab, ipilimumab	Preoperative	UNKNOWN	Hepatocellular carcinoma	2	INTERVENTIONAL	2019–2-12
NCT03337841	Pembrolizumab	Preoperative, postoperative	UNKNOWN	Hepatocellular carcinoma	2	INTERVENTIONAL	2017–11-10
NCT03630640	Nivolumab	Preoperative, postoperative	ACTIVE, NOT RECRUITING	Hepatocellular carcinoma	2	INTERVENTIONAL	2018–10-11
NCT06003673	Tislelizumab, lenvatinib	Preoperative	RECRUITING	Hepatocellular carcinoma	4	INTERVENTIONAL	2023–7-1
NCT05471674	Nivolumab	Preoperative	COMPLETED	Hepatocellular carcinoma; liver cancer	2	INTERVENTIONAL	2020–7-3
NCT05908786	Atezolizumab, bevacizumab, tiragolumab, tobemstomig	Preoperative	RECRUITING	Carcinoma, hepatocellular	1|2	INTERVENTIONAL	2023–12-5
NCT04658147	Nivolumab, relatlimab	Preoperative, postoperative	RECRUITING	Hepatocellular carcinoma	1	INTERVENTIONAL	2021–5-28
NCT04224480	Pembrolizumab	Preoperative, postoperative	ACTIVE, NOT RECRUITING	Hepatocellular carcinoma	1	INTERVENTIONAL	2019–12-10
NCT04727307	Atezolizumab, bevacizumab	Preoperative, postoperative	RECRUITING	Hepatocellular carcinoma	2	INTERVENTIONAL	2021–1-26
NCT03867370	Toripalimab, lenvatinib	Preoperative, postoperative	TERMINATED	Hepatocellular carcinoma	1|2	INTERVENTIONAL	2019–4-26
NCT04954339	Atezolizumab, bevacizumab	Preoperative, postoperative	RECRUITING	Hepatocellular carcinoma	2	INTERVENTIONAL	2021–10-29
NCT04857684	Atezolizumab, bevacizumab	Preoperative	RECRUITING	Hepatocellular carcinoma	1	INTERVENTIONAL	2021–6-18
NCT05440864	Tremelimumab	Preoperative, postoperative	RECRUITING	Hepatocellular carcinoma	2	INTERVENTIONAL	2023–10-26
NCT05701488	Durvalumab, tremelimumab	Preoperative, postoperative	RECRUITING	Hepatocellular carcinoma, hepatocellular cancer	1	INTERVENTIONAL	2023–4-21
NCT03299946	Cabozantinib, nivolumab	Preoperative	COMPLETED	Hepatocellular carcinoma	1	INTERVENTIONAL	2018–5-14
NCT05389527	Pembrolizumab, lenvatinib	Preoperative, postoperative	ACTIVE, NOT RECRUITING	Hepatocellular carcinoma	2	INTERVENTIONAL	2022–9-30
NCT05137899	Atezolizumab, bevacizumab	Preoperative, postoperative	RECRUITING	Hepatocellular carcinoma	2	INTERVENTIONAL	2022–10-18
NCT04425226	Pembrolizumab, lenvatinib	Pre- and post-liver transplant	RECRUITING	Hepatocellular carcinoma	NA	INTERVENTIONAL	2020–8-6
NCT04297202	Apatinib, SHR-1210 (anti-PD-1 inhibitor)	Preoperative, postoperative	UNKNOWN	Hepatocellular carcinoma	2	INTERVENTIONAL	2019–12-1
NCT05185531	Tislelizumab	Preoperative, postoperative	ACTIVE, NOT RECRUITING	Hepatocellular carcinoma	1	INTERVENTIONAL	2022–3-1
NCT05185505	Atezolizumab, bevacizumab	Pre- and post-liver transplantation	RECRUITING	Hepatocellular carcinoma, hepatocellular cancer	4	INTERVENTIONAL	2023–1-30
NCT03916627	Cemiplimab, fianlimab	Preoperative, postoperative	RECRUITING	Non-small cell lung cancer, hepatocellular carcinoma, head and neck squamous cell carcinoma	2	INTERVENTIONAL	2019–7-23
NCT05621499	Sintilimab, lenvatinib	Preoperative	NOT YET RECRUITING	Hepatocellular carcinoma	NA	INTERVENTIONAL	22-Nov
NCT04930315	Apatinib, camrelizumab	Preoperative, postoperative	RECRUITING	Hepatocellular carcinoma	2	INTERVENTIONAL	2021–9-15
NCT05225116	Sintilimab, lenvatinib	Preoperative	RECRUITING	Hepatocellular carcinoma	1	INTERVENTIONAL	2023–1-8
NCT04123379	Nivolumab, BMS-813160(CCR2/5-inhibitor), BMS-986253 (Anti-IL-8)	Preoperative, postoperative	ACTIVE, NOT RECRUITING	Non-small cell lung cancer, Hepatocellular carcinoma	2	INTERVENTIONAL	2020–3-5
NCT05807776	Tislelizumab, lenvatinib	Preoperative, postoperative	NOT YET RECRUITING	Resectable hepatocellular carcinoma	2	INTERVENTIONAL	2023–4-1
NCT04850040	Apatinib, camrelizumab, oxaliplatin	Preoperative	NOT YET RECRUITING	Resectable hepatocellular carcinoma	2	INTERVENTIONAL	2021–5-6
NCT04615143	Tislelizumab, lenvatinib	Preoperative, postoperative	RECRUITING	Recurrent hepatocellular carcinoma	2	INTERVENTIONAL	2020–12-1
NCT04653389	Sintilimab	Preoperative, postoperative	TERMINATED	Hepatocellular carcinoma	2	INTERVENTIONAL	2020–12-26
NCT05578430	Cadonilimab	Preoperative, postoperative	NOT YET RECRUITING	Hepatocellular carcinoma	2	INTERVENTIONAL	2023–1-1
NCT04443322	Durvalumab, lenvatinib	Pre-liver transplantation	RECRUITING	Liver carcinoma	NA	INTERVENTIONAL	2020–9-19
NCT05194293	Durvalumab, regorafenib	Preoperative	RECRUITING	Hepatocellular carcinoma	2	INTERVENTIONAL	2023–6-29
NCT05339581	Pembrolizumab, sintilimab, camrelizumab, tislelizumab, lenvatinib	Preoperative	NOT YET RECRUITING	Liver cancer, hepatocellular carcinoma	NA	INTERVENTIONAL	2022–5-20
NCT04850157	Pembrolizumab, sintilimab, camrelizumab, tislelizumab, lenvima	Pre-liver transplantation	UNKNOWN	Hepatocellular carcinoma	2	INTERVENTIONAL	2021–4-20
NCT05613478	Camrelizumab, apatinib	Preoperative, postoperative	RECRUITING	Hepatocellular carcinoma	3	INTERVENTIONAL	2022–11-1
NCT04521153	Camrelizumab, apatinib	Preoperative	RECRUITING	Hepatocellular carcinoma	NA	INTERVENTIONAL	2021–3-25
NCT05920863	Tislelizumab, lenvatinib	Preoperative	RECRUITING	Hepatocellular carcinoma	2	INTERVENTIONAL	2023–7-1
NCT04888546	Anlotinib, TQB2450 (anti-PD-L1 antibody)	Preoperative	RECRUITING	Hepatocellular carcinoma	1|2	INTERVENTIONAL	2021–4-30

NA, No clear information on the staging of clinical trials on ClinicalTrials.gov.

Xia et al. analyzed 18 patients with resectable HCC undergoing camrelizumab plus apatinib neoadjuvant therapy, then discovered that a high expression of DCs could serve as a predictor for a favorable response to neoadjuvant therapy. They further observed that higher levels of DCs after neoadjuvant therapy were associated with a reduced likelihood of relapse. Additionally, they found that post-perioperative treatment with ctDNA positivity was more prevalent among patients without major pathological reactions, indicating the potential of ctDNA as a predictive marker for early recurrence (126). Ho et al. studied 15 patients with locally advanced HCC receiving neoadjuvant cabozantinib and nivolumab, and observed an enrichment of T effector cells, tertiary lymphoid structures, CD138+ plasma cells, and a distinctive spatial arrangement of B cells among responders compared to non-responders. Specifically, the proximity of B and T cells to proliferative macrophages expressing higher levels of PD-L1 was a notable characteristic of tumors that responded to cabozantinib and nivolumab. Conversely, the proximity of B and T cells to macrophages exerting immunosuppression through arginase-1 was a critical feature of tumors resistant to cabozantinib and nivolumab. These findings suggest the coordinated contribution of B cells to antitumor immunity in HCC (127). Magen et al. identified pathological responses in patients characterized by intratumoral cellular triads consisting of progenitor CD8+ T cells and CXCL13+ CD4+ T helper cells surrounding mature DCs (referred to as “mregDC”) by analyzing of 20 patients with resectable HCC receiving neoadjuvant cemiplimab. Notably, these niches were more prevalent in tumors from responders even before the initiation of treatment (128). Zhang et al. investigated 15 patients with potentially resectable HCC participating in a single-arm, open-label, phase 1 clinical trial of neoadjuvant cabozantinib and nivolumab by utilizing spatial transcriptomics. They found that the TME of responding tumors exhibited the enrichment of immune cells and cancer-associated fibroblasts (CAF) with pro-inflammatory signaling compared to non-responders. They also observed that B cells could serve as the initial trigger for tumor cell killing by cytotoxic immune cells and the recruitment of other effector immune cells. Additionally, HCC-CAF interactions were more prevalent in the responding tumors and were associated with extracellular matrix remodeling (129). Marron TU et al. enrolled 21 patients with resectable HCC (stage Ib, II, and IIIb). This represents the largest reported clinical trial to date of neoadjuvant anti-PD-1 monotherapy for HCC. All patients received neoadjuvant cemiplimab, and 20 patients underwent successful resection. Of the 20 patients with resected tumors, four (20%) had significant tumor necrosis. Three (15%) of 20 patients had a partial response, and all other patients maintained stable disease. The authors believe that the observed pathological responses to cemiplimab in this cohort support the design of larger trials to identify the optimal treatment duration and definitively establish the clinical benefit of preoperative PD-1 blockade in patients with HCC (130). Kaseb AO et al. enrolled 27 patients with resectable HCC. In this phase 2 randomized, open-label study, 13/27 randomized patients were treated with nivolumab and 14/27 with nivolumab plus ipilimumab. 7/27 patients had surgical cancellations, but not due to treatment-related adverse events (AEs). 6/20 resected patients (33%; 3 in each treatment arm) achieved a major pathological response (MPR defined as 100% necrosis (complete response, CR) plus >60% necrosis). 5/6 MPR patients achieved CR. After a median follow-up of 24.6 months (95% CI: 21.6, 34.1), no recurrence was observed in patients with MPR, while 7/14 patients without MPR developed recurrence (131).

The results obtained from the exploration of predictive biomarkers in neoadjuvant HCC were mostly based on the combination of several markers, which differed noticeably from previous findings and highlighted the concept of tertiary lymphoid structures. Current research on TLSs in HCC is primarily retrospective and focuses on prognostic biomarkers, but can provide certain guidance for neoadjuvant clinical trials in HCC. Furthermore, Magen et al. discovered that patients who responded to neoadjuvant cemiplimab already exhibited intratumoral cellular triads prior to surgery (128). In their pathological review of 273 patients who underwent surgical resection for HCC, including both intra-tumoral and non-tumoral tissues, Calderaro et al. found that the presence of intra-TLSs was associated with a reduced risk of early HCC relapse after surgery. Additionally, these intra-tumoral TLSs may indicate the presence of ongoing and effective anti-tumor immune responses (132). In a study involving 462 HCC patients, Li et al. demonstrated an inverse correlation between the presence of TLSs and the risk of early tumor recurrence (133). Moreover, they found that a high density of peritumoral TLSs was associated with a TME characterized by an active immune response and improved patient survival, thus serving as a promising prognostic biomarker for HCC (134).

## Discussion

In the process of summarizing the results of HCC predictive biomarkers, we did not include the results from studies utilizing HCC cell lines and preclinical animal models due to the significant gap in forward translation. Similarly, we did not include results related to anti-drug antibodies, relative dose intensity, and hand–foot skin reactions, because these results are specific to drug usage and do not serve the purpose of being predictive biomarkers for patient selection. Additionally, Albumin–Bilirubin grading and Child–Pugh grading were not assessed, as patients with higher grades tend to have better prognosis even without any anticancer treatment. Due to limitations in time and space, only the classic treatment drugs and approaches used in the history of HCC systemic therapy were included, and many other topics, such as TME subtyping, MAIT cells, bispecific T-cell engagers (135, 136), tumor vaccines, and immune therapies beyond PD-L1 (137), were not discussed.

The exploration of HCC predictive biomarkers is intertwined with the history of advancements in medical technology and drug development. The discovery of pharmacological agents represents the culmination of expertise in forward translation research, leading to an improved understanding of HCC. Thanks to the development of these drugs, the treatment efficacy for advanced HCC has significantly improved, and an increasing number of patients are benefiting from immunotherapies. Although efforts have been made to explore the predictive biomarkers for these therapies, satisfactory markers have not yet been identified. However, relying on one or two biomarkers alone is insufficient for fulfilling the task of HCC predictive biomarkers. Therefore, a shift from previous forward translation to reverse translation research is necessary to validate the results obtained from reverse translation via mechanistic investigations conducted in forward translation. This may be the future’s primary research direction and the most promising path for the discovery of HCC predictive biomarkers.

In summary, no treatment can reverse the progression of HCC. Therefore, in the era of immunotherapy, it is more likely that patients with pre-existing antitumor immune cycles will be identified and their immune responses strengthened by the addition of immunotherapy and anti-angiogenic treatments. This transformation will turn a weak trickle of antitumor immune response into a robust flow, leading to immune normalization both systemically and within the tumor, resulting in tumor attenuation, shrinkage, and improved resolution of macroscopically invisible tumors. In the future, it is possible that leveraging multi-omics technologies and comparing pre- and post-treatment samples of body fluids, blood, LNs, and tumor tissues in well-designed neoadjuvant clinical trials will help identify predictive biomarkers that confirm the “cancer-immunity cycle” (67, 68), “normalizing tumor vasculature” (70), and “normalization of the TME” (71). This approach represents the most promising path for discovering predictive biomarkers for HCC.

## Author contributions

CW: Conceptualization, Writing – original draft. FW: Writing – original draft, Formal analysis, Investigation, Resources. XS: Formal analysis, Writing – original draft, Conceptualization, Supervision. WQ: Conceptualization, Supervision, Writing – original draft, Software. YY: Conceptualization, Supervision, Writing – original draft, Formal analysis. DS: Conceptualization, Writing – original draft, Validation, Visualization. YZ: Conceptualization, Validation, Writing – original draft. JL: Writing – original draft, Data curation, Project administration, Resources, Software. ZF: Writing – original draft, Conceptualization, Formal analysis, Supervision. GL: Conceptualization, Supervision, Writing – original draft. GW: Writing – original draft, Methodology, Project administration, Software.

## Glossary

AFP: Alpha-Fetoprotein
CAF: Cancer-Associated Fibroblasts
cDCs: Conventional Dendritic Cells
cfDNA: cell-free tumor DNA
CR: complete response
CTCs: Circulating Tumor Cells
ctDNA: Circulating Cell-Free Tumor DNA
CTLs: Cytotoxic T Lymphocytes
CTLA-4: Cytotoxic T Lymphocyte-Associated Molecule-4
DCs: Dendritic Cells
DOX: Doxorubicin
ERK: Extracellular-Signal-Regulated Kinase
FDA: Food and Drug Administration
HBV: Hepatitis B Virus
HCV: Hepatitis C Virus
HCC: Hepatocellular Carcinoma
ICIs: Immune-Checkpoint Inhibitors
IHC: Immunohistochemistry
LNs: Lymph Nodes
MAFLD: Metabolic Dysfunction-Associated Fatty Liver Disease
MAIT: Mucosal-Associated Invariant T Cells
MKIs: Multitarget Kinase Inhibitors
MPR: major pathological response
ORR: Objective Response Rate
OS: Overall Survival
PDGFR-β: Platelet-Derived Growth Factor Receptor Beta
PD-1: Programmed Cell Death Receptor-1
PD-L1: Programmed Cell Death Ligand-1
pERK: Phosphorylated Extracellular Signal-Regulated Kinase
PFS: Progression-Free Survival
RTKs: Receptor Tyrosine Kinases
SNPs: Single Nucleotide Polymorphisms
TACE: Transarterial Chemoembolization
TDLNs: Tumor-Draining Lymph Nodes
Tex: Exhausted CD8+ T Cells
TILs: Tumor Infiltrating Lymphocytes
TKI: Tyrosine Kinase Inhibitor
TLSs: Tertiary Lymphoid Structures
TME: Tumor Microenvironment
Tregs: Regulatory T Cells
TTP: Time to Progression
VEGFR: Vascular Endothelial Growth Factor Receptors.
